# Evaluating a dementia learning community: exploratory study and research implications

**DOI:** 10.1186/s12913-018-2894-3

**Published:** 2018-02-05

**Authors:** Rod Sheaff, Ian Sherriff, Catherine Hagan Hennessy

**Affiliations:** 1 School of Government, Plymouth University, Drake Circus, Plymouth, PL4 8AA UK; 2 Academic Partnership Lead for Dementia, Plymouth University Peninsula Schools of Medicine and Dentistry, Plymouth University, Drake Circus, Plymouth, PL4 8AA UK; 3 Faculty of Social Sciences, Stirling University, Iris Murdoch Building, Stirling, FK9 4LA UK

**Keywords:** Dementia, Dementia Learning Community, Logic model, Plan-Do-Study-Act, Unplanned admissions, Residential care, England

## Abstract

**Background:**

Access times for, the costs and overload of hospital services are an increasingly salient issue for healthcare managers in many countries. Rising demand for hospital care has been attributed partly to unplanned admissions for older people, and among these partly to the increasing prevalence of dementia. The paper makes a preliminary evaluation of the logic model of a Dementia Learning Community (DLC) intended to reduce unplanned hospital admissions from care homes of people with dementia. A dementia champion in each DLC care home trained other staff in dementia awareness and change management with the aims of changing work routines, improving quality of life, and reducing demands on external services.

**Methods:**

Controlled mixed methods realistic evaluation comparing 13 intervention homes with 10 controls in England during 2013–15. Each link in the assumed logic model was tested to find whether that link appeared to exist in the DLC sites, and if so whether its effects appeared greater there than in control sites, in terms of selected indicators of quality of life (DCM Well/Ill-Being, QUALID, end-of-life planning); and impacts on ambulance call-outs and hospital admissions.

**Results:**

The training was implemented as planned, and triggered cycles of Plan-Do-Study-Act activity in all the intervention care homes. Residents’ well-being scores, measured by dementia care mapping, improved markedly in half of the intervention homes but not in the other half, where indeed some scores deteriorated markedly. Most other care quality indicators studied did not significantly improve during the study period. Neither did ambulance call-out or emergency hospital admission rates.

**Conclusions:**

PDSA cycles appeared to be the more ‘active ingredient’ in this intervention. The reasons why they impacted on well-being in half of the intervention sites, and not the others, require further research. A larger, longer study would be necessary to measure definitively any impacts on unplanned hospital admissions. Our evidence suggested revising the DLC logic model to include care planning and staff familiarisation with residents’ personal histories and needs as steps towards improving residents’ quality of life.

## Background

### Policy

After the 2008 financial crash, austerity conditions and policies have made hospital costs and overload an increasingly salient health policy issue in many countries. In the UK, reducing hospital bed use became a pillar of fiscal control, since 67.5% of NHS costs arise from hospital care [1]. UK policy makers attributed the rising demand for hospital care above all to unplanned admissions for older people. It has been known since the 1960s that older people, especially frail older people, used a disproportionately high share of NHS beds [2–4] partly because hospitals were often providing essentially residential care in the absence of alternative provision [2]. More recently, UK policy makers have attributed part of this bed use to the increasing prevalence of dementia, which became a prominent health policy issue and remit of one of the Prime Minister’s three special working groups on healthcare.

For people who require care and cannot care for themselves (or be cared for) at home, even *clinically* ‘unnecessary’ hospital admissions can only be avoided if non-hospital (‘community based’) residential care is available for them. The NHS has therefore became increasingly reliant on intermediate care, nursing and care homes as means for containing demand for in-patient care. Meanwhile local government budgets (the largest source of finance for residential care in the UK) have been cut, so that families often have to contribute to care home costs. First admission to residential care is tending to occur later in the progression of dementia making care homes’ case-mix more dependent and complex. In 2012 some 320,000 (80% of) people residing in UK care homes had dementia or severe memory problems. Only 41% of family members thought that these settings gave the person with dementia a good quality of life [5]. Furthermore the workforce in UK care homes is very casualised, with high rates of staff turnover. There are no official requirements for staff working in care homes to have any prior training [6–8]. 86% of care home staff felt that providing care to people with dementia was challenging; almost half the staff respondents reported that they want more training in this area [5]. It has become increasingly necessary to develop care homes’ capacity to anticipate and prevent health deterioration which might otherwise require unplanned hospital admission. Other health systems face similar issues.

### Dementia learning community logic model

Although the UK currently has no regulatory requirement for residential care staff to be trained in dementia care, its government has recently set targets for such training [6, 7]. At the time of this study, the immediate training need was therefore for some training at all, starting at the most basic level. Accordingly, quality improvement programmes including specific strategies for dementia are developing. One influential example was the county-wide, multi-agency Dementia Link Worker (DLW) scheme in Gloucestershire, which reportedly reduced referrals to NHS services [9]. One component of the scheme - a training programme (‘Dementia Leadership Award‘) aimed at care home owners, managers and clinical leads - was reported to produce the culture change necessary for understanding and supporting DLWs’ role [10]. This finding reflects the growing importance of leadership as a concept in dementia education when resource shortages constrain the extent of staff training available and those trained must act as change agents to realise the benefits of this education in practice [11]. Cultivating leadership, increasing staff knowledge and support, and networking informational resources are all elements of interventions involving ‘dementia champions’, a role which has been employed in various forms in both acute and residential care settings in the UK and elsewhere [12–16]. Reports on the use of dementia champions in residential care have described barriers and facilitators to the uptake of activities arising from this role [14, 15]. However studies which quantify the outcomes of care home initiatives using dementia champions are until now lacking.

Staff in an NHS Trust in England wished to meet similar training needs in their own locality, so as to stimulate organisational changes in care homes, thereby improving care home services and reducing demands on NHS services. To devise a way of doing so they first drew upon published studies (then scarce), press reports (professional periodicals, BBC, on-line) and site visits to other similar projects. They then undertook an initial qualitative assessment in two care homes in their own locality (which were among those that subsequently piloted the DLC intervention described below). This assessment characterised the homes’ resident population with dementia; staff qualifications (NVQ/QCF); the facilities’ previous dementia training work, its delivery mode and location; care home managers’ views of the principal issues in dementia care provision; and their ideas for conducting the training and supporting staff participation during an intervention to address these issues. The NHS staff combined contents from these different sources into an 8-hour multi-module training programme which was developed to include group sessions carried out by the project’s dementia learning facilitators (NHS dementia nurse specialists) covering the nature of dementia; principles of communicating with a person with dementia; influence of the environment upon dementia care; person-centred care; care planning and end-of-life care; the Mental Capacity Act and its implications for dementia care; dealing with challenging behaviour; and creating and managing organisational change using Appreciative Inquiry (a method that focuses on exploiting an organisation’s existing strengths [17]).

The training was to be the first step in a more complex intervention, the ‘Dementia Learning Community' (DLC) model. Like any such intervention the DLC model embodied a specific ‘programme theory’. Alongside normative assumptions about what policy outcomes are desirable it contained a ‘logic model’. In practice often partly explicit and partly implicit, such a ‘theory-in-use’ [18] logic model specifies the intervention’s activities and resources, and what outputs and outcomes those who make the intervention assume will result [19]. The DLC logic model was as follows:

1. Dementia learning facilitators identify in each care home a ‘dementia champion’ who is then trained, with other staff, in dementia awareness and how to conduct ‘Plan-Do-Study-Act’ (PSDA) cycles to improve quality of life [20–22]. Subsequently the learning facilitators regularly visit each dementia champion and use networking activities (teleconferences; web-based forum; monthly team awards; newsletter; annual conference) to help the dementia champions to develop leadership skills and confidence; clarify staff roles in the homes; promote best practice in person-centred care; improve care planning; and enhance the care environment.
2. These activities would

- increase staff job-satisfaction and self-development; and impart knowledge, attitudes, behaviours and skills associated with best practice in dementia care.
- initiate and sustain PDSA cycles including care audits and quality improvement and spread activities in each care home.

3. Together the impacts at step 2 above would result in everyday care home work routines increasing residents’ well-being.
4. Longer term, the improved work routines would raise the residents’ health-related quality of life. Better planning of care and better monitoring of residents’ condition would lead to earlier observation of emerging health-related problems, and therefore to remedial action (i.e. secondary prevention) to prevent these problems developing.
5. Raising their health-related quality of life would reduce residents’ need for ambulance call-outs and unplanned (emergency) hospital admissions.

The facilitators’ follow-up sessions with the dementia champions converted a training programme into a PDSA intervention and were the rationale calling it a ‘learning community’. The DLC model was therefore novel in linking attempts at culture change in care homes strongly to the development of PDSA activities. Such methods for improving service quality are extensively researched in hospitals but not care homes, so findings from the initial evaluative research which this paper reports may have wider interest and application.

**Fig. 1:**
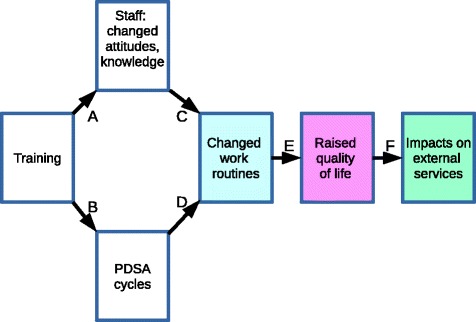
DLC logic model

The above logic model is what we tested in this evaluation. Figure 1 shows the main causal links (labelled ‘A’-'F’) which the DLC logic model assumed.

Links A, B and D apply only to DLC sites. The more generic links C, E and F would be found in both there and elsewhere, but with stronger effects in DLC sites. In practice each link is likely to be confounded and one would expect the effect of the initial training to become attenuated at each step. Training alone has more impact on care workers’ knowledge of dementia than on their coping styles [23] hence may not be enough to raise quality of life for people with dementia [24]. Indeed training in safeguarding vulnerable adults and longer time working in the sector may *reduce* trainees’ confidence, possibly because better-informed staff become less complacent that residents’ needs are being properly safeguarded, although the greater the apparent deficiencies in care, the more confident trained staff become about reporting them [25]. Dementia Care Mapping (DCM: see below) may in itself help reduce staff burnout [26] so that improved service quality feeds back into improved staff morale. Person-centred care appears to reduce agitation [27] and use of antipsychotic drugs [28] in people with dementia, and to improve staff recognition of cognitive impairment in residents [29]. Research on the effects of quality improvement and spread methods, particularly PDSA cycles [30] mostly concern acute hospitals. Studies set in services for older people with cognitive impairment are scarce but also tend to report positive outcomes [21, 31, 32]. However residential care work routines are not the only influence on residents’ quality of life, and the latter is not the only determinant of unplanned hospital admissions [33]. Similar qualifications apply to studies of the effects of networking in sustaining changes in work routines (e.g. showing that inter-nursing home collaboration improves uptake of pain-management methods [34]) or in improving mental health care as rated by patients and carers [35].

### Research questions

In the absence of studies evaluating the DLC logic model, we aimed to make a preliminary exploratory attempt to evaluate it empirically. It was exploratory in that we aimed not only to assess how far the model had been implemented and its effects, but in doing so also to elicit, test and refine the underlying ‘theory-in-use’ logic model of how a DLC works. In that way we could assess whether, or with what modifications, the DLC model might be reproducible elsewhere. By exploring the effects and their magnitudes we also could lay the basis, if the findings warranted it, for a more definitive and detailed evaluation that would supplement the evidence base for care for people with dementia. We therefore addressed the following research questions. Each corresponds to a stage in the logic model.

In the care homes that participated:

1. Were the initial training and facilitation implemented as specified?
2. What was the impact on staff job satisfaction, knowledge and attitudes about dementia and confidence in providing care to persons with dementia?
3. What PDSA activities resulted?
4. What ensuing changes in work routines and therefore resident well-being could be attributed to the impacts on staff job satisfaction, knowledge, attitudes about dementia and confidence and/or to the PDSA activities?
5. How did measured quality of life change for residents?
6. What were the impacts on ambulance call-outs and emergency hospital admissions?

## Methods

### Design

Since any policy or organisational intervention tacitly applies a logic model [36], a realist approach is to make that logic model explicit and expose it to evidential testing, so that it can be revised as necessary and thereby strengthened. Because we aimed to evaluate the logic model on which its inventors had implicitly based the DCL, realistic evaluation [26] was the study design of choice, not least because that design focuses attention on understanding the context in which an intervention is carried out and how that context (also) influences the observed outcomes [27]; in the present case, determining how the DLC intervention worked in care environments typified by a low-paid, casualised work force with high turnover [8]. We therefore adopted a multi-method realist evaluation [37] research design comprising:

1. An implementation component investigating the logic model’s implementation structures [38], what factors facilitated or impeded implementation, or affected its outcomes [39], from the initial training through to the follow-up work to support any ongoing PDSA cycles. This component addressed research questions 1, 2 (partly), 3 and 4 (partly).
2. An impact component testing, link by link, whether each link in the logic model appeared to be present, addressing research questions 2 (partly), 4 (partly), 5 and 6.

A necessary preliminary to both was to elicit the logic model which those who designed and implemented the DLC were using. We did that through discussions, interviews and e-mail correspondence with them, by context-analysing project documentation and websites, and content-analysing research publications that the project implementers said they had used. Some of these materials are cited, and the resulting logic model reported, above.

### Setting and sampling

Both components used the same settings, but the implementation component applied only to the DLC homes in the sample. All 23 study homes were privately owned, representing about a quarter of the care homes in one English local authority. They volunteered to participate in the understanding that those selected as controls could also become DLC sites once the study finished. We randomly allocated homes to intervention (DLC) and control groups by file sampling the list of volunteer care homes (Table 1). Achieving approximately equal numbers of residents in each arm required 23 care homes (= 10 controls + 13 DLC).

**Table 1:** Control and DLC sites

	Control	DLC
N (homes)	10	13
Ownership	4 corporate; 6 owner-managed	10 corporate; 1 owner-managed; 1 religious; 1 charitable.
Residents	330 (range 20–51)	288 (range 14–46)
Staff	245 (range 17–50) [*]	298 (range 11–53)
Mean staff wte per bed	0.82 (range 0.61–1.03)	1.02 (range (0.59–1.58)

[*] For 9 homes. No data from one owner-managed home

At the outset we compared residents’ baseline DEMQOL, QUALID and DCM scores (see below) between the DLC and control sites to check whether the former already appeared to be giving higher-quality care and/or to have residents in better health (in terms of dementia), but there were no significant differences at that stage.

### Implementation component

To examine whether, and if so how, DLC training had created links A, B and D we used qualitative methods, taking an ethnographic approach [40, 41] to discover how the dementia champions in each DLC home were recruited and trained, any PDSA activities, their practical consequences, what was being done to sustain them, and any difficulties or resistance. Training session participants completed review questionnaires, from which we collated their responses about what they had learned and what they intended to do as a result. We interviewed four of the dementia champions and analysed all the network facilitators’ contemporary field-notes of their four-weekly meetings with each dementia champion. We analysed these data inductively and thematically [42], seeking patterns across the DLC homes in how the logic model links were, or were not, established in practice.

### Impacts component

#### Measures

We treated each link as having an antecedent (as the case may be, initial DLC training or the later changes it was intended to trigger) and a consequent (impact). All measures except (1) and (6) below were therefore used twice, as the consequent of one link and the antecedent for the next.

1. DLC Intervention: completion of training sessions (yes/no).
2. PDSA cycle: evidence of PDSA activity (yes/no).
3. Changes in staff attitudes, knowledge of dementia and morale were assessed by changes in scores on the: Dementia Attitudes Scale (DAS) [43]; Approaches to Dementia Questionnaire (ADQ) [44]; and the English version of the Swedish Satisfaction with Nursing Care and Work Assessment Scale (SNCW) [45], in which *lower* scores indicate greater satisfaction with, or a more positive attitude towards, work). As a proxy for staff morale and for constraints on relational continuity of care, we measured sickness leave rates per member of staff per home for the year before and the year after the start of the DLC intervention.
4. Immediate effects of changed work routines on residents were measured by changes in the Well- and Ill-Being (WIB) scores used in Dementia Care Mapping (DCM) [46, 47], interpreting increased scores as evidence of changed work routines. We used a two-hour observation frame which is within the range for previously published evaluations [47] and sufficient for psychometric stability [48]. We regarded DCM as a measure, not as an intervention in its own right for making staff more resident-centred in their work [27].
5. Changes in dementia-specific measures of residents’ quality of life: We used the Global Deterioration Scale (GDS) [49] to decide whether residents’ quality of life should be measured using DEMQOL [50] (a self-response tool) or, for residents with a GDS score of 4 or above (mild dementia/moderate cognitive impairment) the Quality of Life in Late-Stage Dementia (QUALID) scale [51]. (DEMQOL-Proxy, an otherwise suitable measure, was not feasible because few residents had non-staff carers to hand.) We also measured the change in number of residents with records of an end of life discussion, end of life care plan, an advanced care plan and a completed treatment escalation plan (TEP) form.
6. External impacts: changes were measured by the proportionate change in homes’ annualised rates, per resident, of:

- Ambulance call-outs, as reported in the homes’ administrative records.
- Hospital admissions: both unplanned and planned, using routine administrative data from the local Clinical Commissioning Group.

Because the care home (site) was the unit of analysis, we aggregated individual-level measures to yield mean scores per home, which also removed any clustering in individual-level scores and compensated for the different sizes of homes.

#### Sampling

Before starting fieldwork we estimated from published reports [52, 53] that a sample of 384 residents (192 + 192) was necessary to give a 90% chance of detecting a 5% superiority of outcome in the DLC programme, significant at the 5% level, using DEMQOLv4 [50] as our quality measure or, using QUALID, 632 residents (316 + 316). In the event achieving even the smaller sample size was difficult because of the high drop-out rate due to the natural progression of dementia, reducing the eventual pre- and post-intervention dataset to 246 residents. Furthermore, GDS rating showed that only 27 residents in the post-intervention dataset were suitable for the DEMQOL measure of quality of life, leaving QUALID as the default.

#### Analyses

We tested each link of the logic model in two steps.

First, if a link were present, changes in its antecedent(s) would be associated with changes in its consequent(s). Since DLC training was their antecedent, links A, B and D would be found only in the DLC sites, so only data from those sites could be used to test whether those links existed. The remaining links were generic and should, the logic model implied, exist in all sites. So to bring more data to bear, we tested for those associations across all the study sites.

Second, each link would produce a bigger change in its consequent (staff characteristics, work routines, quality of life or the external impacts, as the case might be) in the DLC sites than controls. For staff characteristics, work routines and quality of life we had only one data-point available before and one after the DLC intervention. We therefore made those comparisons by testing cross-sectionally for any differences in the proportionate change (i.e. the mean post-intervention score divided by the mean pre-intervention score per home) in these measures between DLC and non-DLC sites. For the external impacts, routine administrative data allowed us to make difference-in-differences analyses. Because sites’ mean duration of participation in the DLC was 12.3 months (range 11 to 14 months) we took the preceding 12 months (October 2013–September 2014) as the matched control period. For all statistical tests (Wilcoxon signed rank, linear regression) we used version 3.3.1 of the R software with a declared significance level of *p* ≤ 0.05. (Relative and absolute risks or risk reductions are not relevant to these tests. Confidence intervals are not relevant to Wilcoxon tests.)

### Ethics

All these privately-owned homes fell outside the remit of the NHS and local authority research ethics approval systems. We therefore obtained ethical approval from Plymouth University Research Ethics Committee on 21st February 2014 (Reference PU13/14–216), as a condition of which informants and care homes are pseudonymised below. Informed consent was obtained from all participating care home staff. Consent procedures for residents accommodated their capacity to provide permission to participate [54]: for those with mild to moderate dementia, their willingness to take part in the research was confirmed on an ongoing basis, while for those with severe dementia, consent was sought from a family member or other available proxy with designated authority to provide consent. We obtained individual consent (including consent to audio-record interviews) from individual care home managers and staff members.

## Results

After reporting response rates we present our results for each link in the logic model. For brevity we present only overall scores except where necessary to interpret or disambiguate the results.

DLC trainees’ response rate was 100%. For the staff survey (all sites) it was 202/603 (33%). Staff sickness and turnover data for were available for 18/23 sites (78%). Pre- and post-intervention Dementia Care Mapping (DCM) data were obtainable for 15/23 sites (65%), of which 5 were controls and 10 DLC sites. QUALID data were obtained for 246 residents, i.e. 38% of the study homes’ total population. Data on Treatment Escalation Plans were obtained for 201 residents (31%). Ambulance data for ‘See & Treat’ and ‘See & Convey’, but not for ‘Hear & Treat’, call-outs were available for 21/23 sites (91%). 62 (i.e. 19%) of the initial 332 residents in the study had no formal diagnosis of dementia at the start of the evaluation, but 47 of those 62 had a GDS score of 4 or above, indicating ‘deficits … clearly manifest in a detailed clinical interview … subjects … who enter this fourth stage almost invariably manifest subsequent deterioration characteristic of dementia of the AD type’ [55]. Two DLC sites dropped out, one because a local authority had concerns about quality of life there and ceased referrals to it. The other changed ownership. Testing for the presence or strength of a given link is only possible, however, for the sites which supplied data about both its antecedent and its consequent, so the amount of usable data for each link was less, as reported below.

### Link a: Training and impacts on staff

The same training took place in all DLC sites, and they all implemented it as planned. Table 2 shows the main training outcomes that participants reported.

**Table 2:** Self-reported training outcomes

Better prepared for dealing with aggressive resident behaviour	13
Confidence building	5
Learn about end of life care	1
Communicate better	12
Understanding dementia [named specifically]	108
‘Awareness’, ‘knowledge’, ‘learning’ [topic unspecified]	102
No response	40
Total	282

Dementia champions were recruited by asking the care home managers to identify volunteers for the role, which all except two managers did. In default these two managers took on the dementia champion role themselves. As their intentions for working differently in future, trainees mentioned listening to and observing residents more (staff from sites A,F,J,M,U,W); learning more about the residents as individuals from documented information, other staff and residents themselves (sites A,B,F,I,J,R,U); interacting more with residents and spending more time with them (sites A,B,G,J,I,M,Q,R,S,W); helping fellow-workers work more as a group and communicating more with them (sites B,F,G,J,I,Q,W) e.g. by making better use of the home’s communication book. A few mentioned giving more ‘*person centred*’ care (sites G,M,S) or being more compassionate and tolerant (sites B,G). One, more modestly, intended to *‘stop annoying the residents’* (site S). Only one trainee asserted that there was ‘*nothing*’ he/she wanted to do differently.

As the logic model predicted, DAS and ADQ total scores rose in the DLC sites and the SNCW total scores fell. However the training mostly did not appear to make the staff characteristics change *further* in the predicted direction in the DLC than in the control sites. Mean DAS total score rose by 2.18 in the DLC group but by 4.39 in the control group, over a scale 120 points long. The difference was not statistically significant (Wilcoxon rank sum W = 57, *p* = 0.86). Neither was the difference for mean ADQ total score, which fell by 0.76 in the intervention group and rose 0.5 in the DLC sites, on a 76-point-long scale (W = 41,*p* = 0.42). Mean SNCW total score fell 7.53 points in the DLC sites but rose 0.56 points in the control sites, on a scale 128 points long, a difference which was statistically significant (W = 65,*p* = 0.045), because of decreases (separately not quite statistically significant) in the Quality (W = 62.5, *p* = 0.07) and Workload (W = 63, *p* = 0.06) components of SNCW. The Cooperation (W = 45.5, *p* = 0.47), Development (W = 58,*p* = 0.14) and Patient Knowledge (W = 60.5,*p* = 0.09) components of SNCW showed no significant difference. Mean sick days per staff member per year significantly decreased by 1.35 in the DLC sites but increased by 0.42 in the control sites (W = 54, *p* = 0.05). We found no significant difference in staff turnover.

### Link B: Training and PDSA cycles

The initial training did trigger cycles of PDSA activity in all homes that participated. Partly the dementia champions expressed their aims for the PDSA cycles in general, aspirational terms, most often *‘To show us the right path regarding the dementia experience’* (site J; and, differently worded, sites F, I,Q,R,S,U,W). ‘*Person centred* (‘*individualised*’, ‘*personalised*’) *care*’ was mentioned in sites G,I,J,Q,R,S and W, and care home culture by the dementia champions for sites A, B and M. However, the dementia champions also stated more concrete, practical aims for their PDSA activity. In descending order of frequency, these aims were: to find out more about the home’s residents in order to inform staff interactions with them (sites G,I,M,S,U,W) or, more concretely, by producing one-page resident profiles or ‘This is me’ notes (sites A,B,F,I,Q,R). A second set of planned activities concerned environmental enhancement for residents, including reviewing care routines and making them more person-centred. These activities included the use of memory boxes (site B), residents helping themselves to vegetables at mealtimes (site S); having books, magazines, newspapers, games (cards, dominoes) for all residents to use when they wanted (site U), enabling residents to have personal items to hand (site U) or having their room set up as they liked (site U), and having assisted baths first thing in morning (site G). Staff received further training in dementia awareness at sites J,M,R and W.

### Link C staff characteristics and immediate effects of work routines

Initially the study sites had generally low overall Well- and Ill-Being (WIB) scores. Only six sites scored ≥2.5, and only three of them (B,U,V) met the DCM standards across all categories. At the end of the scheme (approximately one year), five DLC homes showed marked changes in their WIB scores but another five did not (Table 3).

**Table 3:** WIB score changes

Site	Status	PRE Mean WIB	POST Mean WIB	Change
D	C	1.10	0.90	−0.2
H	C	0.73	0.7	−0.03
O	C	0.95	0.94	−0.01
K	C	1.02	1.2	0.18
V	C	0.66	0.85	0.19
A	DLC	0.89	0.00	−0.89
B	DLC	1.80	1.25	−0.65
I	DLC	0.96	0.87	−0.09
S	DLC	0.49	0.54	0.05
M	DLC	0.88	1.14	0.26
R	DLC	1.15	1.95	0.8
F	DLC	1.34	3.21	1.87
U	DLC	1.06	3.01	1.95
Y	DLC	0.03	2.11	2.08
W	DLC	0.31	2.61	2.3

*PRE* pre-intervention, *POST* post-intervention. *C* Control site, *DLC* intervention site

In sites A,B,I,M and S the changes in WIB scores were within the same range as those for the control sites, i.e. they either increased by less than 30% or fell. Indeed DLC sites A and B showed larger falls in WIB score than any of the control sites. In aggregate there was no significant difference between the Low-WIB DLC sites (A,B,I,M and S) and the control sites. A second group of sites (F,R,U,W and Y) showed another pattern. Their WIB scores rose by between 70% and 742%, a statistically significant mean increase of 1.8 points (W < 0.1, *p* < 0.01). For short we label the two groups ‘High-WIB’ and ‘Low-WIB’ sites. It therefore appeared that either or both of links C and D were present in five ‘High-WIB’ sites, and stronger there than in the controls.

To test whether link C was present, we tested for association between the measures of staff characteristics in the DLC sites, and in particular for the High-WIB sites taken alone, and the respective WIB Scores. Taking all the DLC sites together, we found no association between the proportionate changes in DAS total score and WIB score (W = 52, *p* = 0.86) or ADQ total score and WIB score (W = 66, *p* = 0.47). The proportionate change in WIB score was however associated with proportionate change in SNCW score, and negatively as predicted (SNCW being reverse-scored) (W = 97, *p* < 0.01).

Of the presumed antecedents of link C, ADQ and ADS scores (of staff attitudes and knowledge) did not significantly differ between the DLC and the control sites, but SNCW scores did (W = 65, *p* = 0.05), both for all DLC sites and the High-WIB sites taken separately (W < 0.01, p < 0.01).

### Link D: PDSA activity and immediate effects of improved work routines

We found evidence (both interviews and physical artefacts) at the DLC homes that knowledge from the training sessions had been used to initiate post-training PDSA activities directed at improving working practices there. At the High-WIB sites (F,R,U,W,Y) PDSA activities focused on: elaborating residents’ care plans using information gathered at the training session and subsequently (site U); periodic staff meetings about residents (site F); designating key-workers responsible for particular tasks and/or residents (site R); setting up routine team meetings so as to re-iterate and sustain future PDSA cycles (site Y); and training staff by letting them experience what life as a care home resident is like (e.g. being fed by someone else, wearing incontinence pads etc.) (site W). However PDSA cycles in low-WIB homes initiated many similar activities. Sites A and I identified key-workers responsible for particular tasks and/or residents, Sites I and S initiated routine team meetings to sustain future PDSA cycles, since *‘You can learn all the time about dementia’* (Dementia champion). Site B did small-scale initial testing to see if new ideas worked. Homes M and S initiated planning morning activities for residents. Two homes for which we did not have before-and-after WIB data also reported similar post-training PDSA activity. Periodic staff meetings about residents were introduced at sites G and Q. In addition site G identified key-workers responsible for particular tasks and/or residents, established routine team meetings so as to sustain future PDSA cycles, undertook small-scale initial testing to see if ideas worked, and introduced planned morning activities for residents.

So far as we are aware, data from PDSA activities were not usually documented. Neither, therefore, were such data re-used over time. PSDA cycles were predictively-based only in the sense that participants anticipated certain broadly-defined outcomes from the ‘Do’ phase (e.g. that residents would become happier). Thus PDSA cycles were implemented, but compliance with the PDSA model [22] was patchy. There was no PDSA activity in the non-DLC sites.

What differentiated the high-WIB and low-WIB sites was not *whether* PDSA cycles followed the initial training nor, mostly, what the contents (foci) of the PDSA cycles were. So perhaps other factors not recognised in the logic model, such as the organisational character of High-WIB homes, were responsible instead. High-WIB homes tended to be larger (a mean of 29 beds versus 25.8) and have a higher staffing ratios (1.16 wte staff per resident versus 0.93) but in our data these differences were not statistically significant. High-WIB and Low-WIB sites did not differ in terms of the aspirations stated in the post-training questionnaires and follow-up sessions, home location or ownership type. The only resistance to DLC activity was in Low-WIB sites. In one, the manager reported that some staff were suspicious and defensive about the DLC and would not complete residents’ ‘This is me’ documents. That and another Low-WIB site had difficulty recruiting dementia champions. The absence of link C leaves only PDSA activity (link D) to explain the increased WIB scores in the five ‘High-WIB’ DLC sites, but apart from eliminating home location, size, staffing and ownership the data available to us did not reveal what other contextual differences between High-WIB and Low-WIB homes enabled PDSA activities in the former to change working practices sufficiently to raise their WIB scores.

### Link E: Effects of improved work routines and quality of life

At baseline, not one site scored better than the mid-point of the QUALID scale. Taking DLC and control sites together, change in WIB score was associated with proportionate change in total QUALID score (W = 155, *p* = 0.03) and in each QUALID component, although not in the High-WIB homes taken alone. Comparing DLC and non-DLC sites, total QUALID scores fell in both intervention and control homes, by a mean of − 1.68 and − 0.57 respectively for a 44-point scale, but the difference was not statistically significant (W = 39, *p* = 0.23). We found the same pattern for each separate component of QUALID. We also compared only the High-WIB homes with the controls, but again found no significant differences. Considering the high mortality among this population it was striking how few end-of-life care plans there initially were; 42 in the control and DLC homes combined (646 beds). There was no difference between DLC and non-DLC homes in the change in frequency of use of end-of-life care discussions, care plans, or TEP forms.

### Link F: Quality of life and external impacts

Contrary to the logic model, improved QUALID scores were, across all sites, associated with *increased* (not reduced) ambulance call-outs (by an average of 1.6 call-outs per year per home) (W = 343, *p* = 0.03). Comparing all DLC sites with the controls also showed no significant difference in the change in rate of ambulance call-outs (W = 70, *p* = 0.06). In respect of ambulance call-outs, link F was not present.

Across all the sites, changes in QUALID total scores were not associated with changes in the rate of all admissions (W = 373, *p* = 0.08), nor with changes in the rates of emergency admissions specifically (W = 295, *p* = 0.3). At the start of the study DLC sites already had lower admission rates (both planned and unplanned) than the control sites. Nearly all hospital admissions (374/389, 96%) from all the homes were as emergencies. During the project the number of admissions per bed per year did fall in the DLC sites (by 20% for all admissions and 27% for emergencies) but since admissions also fell in the control sites the difference-in-differences analysis showed that for all admissions the ‘treatment’ (i.e. DLC) effect was negligible (δ approaching zero). For emergency admissions it was in the predicted direction but small (δ = − 0.3 emergency admissions per bed per year), and still not statistically significant (*p* = 0.29). For hospital admissions too link F was absent.

## Discussion

Our methods assumed that no confounding change occurred during the DLC project, and no ‘contamination’ of the non-DLC sites with DLC work routines, even though DLC and control sites were often nearby and staff turnover (hence transfer between workplaces) was frequent. Also our methods assumed a comparable case-mix during the two years of the study, and between homes, despite high mortality among residents. Having volunteered as DLC sites, one might expect the study homes to be if anything more motivated than other care homes to implement and exploit the DLC model. Comparing the above results with those of an initial pilot analysis of the external impacts six months into the study, it was noticeable how rapidly the *p*-values fell towards significance as the quantity of data increased, raising the question of whether a longer evaluation might yield results more favourable to DLC. Evaluations of other ‘culture change’ interventions in residential long-term care suggest the importance of allowing interventions enough time to mature or ‘bed down’ before evaluating them [56]. Our results also suggested that if the observed changes in emergency admissions were indeed due to the DLC, they took over six months to appear.

As reported above, ADQ and ADS scores (of staff attitudes and knowledge) did not significantly differ between the DLC and the control sites, but SNCW scores did. We also found that the location, size, staffing and ownership of High-WIB homes, did not appear to explain why Link D, beween PDSA activity and immediate effects of improved work routines, differed between high-WIB and low-WID sites. These results suggest revising the logic model, and in particular re-interpreting link C.

## Conclusion

### Summary results

We aimed to make a preliminary, exploratory empirical evaluation of the DLC and its underlying logic model. In summary we found equivocal evidence for the presence of links A,B,C,D and E, and none for link F (Table 4).

**Table 4:** Empirical status of DLC logic model

Link	Antecedent	Consequent	Measure(s) of consequent	results	
				Link found?	Consequent (‘effect’) stronger in DLC?
A	DLC training (DLC sites only)	Staff characteristics	DAS, ADQ, SNCW	Yes	No for DAS, ADQ and parts of SNCW. Yes for SNCW Development and Patient Knowledge components.
			Sick-leave	Yes	Yes
			Turnover	No	No
B		PDSA cycles	Reported/not reported	Yes	Yes
C	Staff characteristics	Immediate impact of work routines	DCM WIB scores	Partly: for SNCW but not DAS, ADQ.	No
D	PDSA			Yes, but ‘High-WIB’ homes only	Yes, but ‘High-WIB’ homes only
E	Immediate impact of work routines	Quality of life	QUALID	Yes	No
			End-of-life care discussion	No	No
			End-of-life care plans	No	No
			TEP decision forms	No	No
F	Quality of Life	External impacts	Ambulance call-outs	No	No
			Emergency admissions	No	No

Our impact and implementation data both suggested that any effects which the DLC training had upon staff characteristics (link C) came through encouraging staff to know more about their residents and improving staff morale (‘Development’) rather than by changing knowledge or (other) attitudes. This is consistent with other studies demonstrating that the effects on practice of dementia education interventions with residential care staff are best achieved when didactic training is accompanied by experiential learning and support [57]. PDSA activities appeared to be the more ‘active ingredient’ in the DLC programme, but compliance with the PDSA model was patchy. The contrast between High-WIB and Low-WIB DLC sites suggested that care improvement depended on *how* a home implemented its PDSA activity, raising the contextual question of what the High-WIB homes did differently to the other DLC sites (and the controls), for example in terms of sustaining the cycles long-term and adapting them [22]. Finding staff resistance raises questions about what factors affect fidelity of implementation of PDSA activities in residential care. Our results confirmed that one should expect any effects of the DLC would weaken through successive links of the logic model.

### A revised logic model

**Fig. 2:**
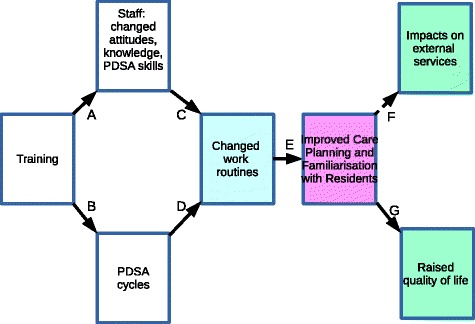
Revised DLC logic model

Nevertheless our findings also show the feasibility of implementing the DLC model, what effects can be expected from it over what timescale, and – subject to further research (see below) – which contexts may help produce those effects. Among other things, the training and PDSA activities stimulated not narrowly clinical monitoring so much as staff familiarisation with residents’ present state, background, personal interests, preferences and needs; and improved care planning. In turn these activities improved residents’ quality of life. The original logic model did not clearly differentiate these sequential events. Above we infered that link C in the original logic model required reinterpretation. The relevant ‘knowledge and attitudes’ appeared to be attitudes towards staff development and knowledge about residents (part of what the SNCW records). Since PDSA cycles appeared to be a key ‘active ingredient’ in the DLC model, knowledge and practical skills in carrying them out should also be considered part of link C. The more immediate and reliable outcome of DLC model lay in using PDSA cycles to improve health-related quality of life for care home residents with dementia. Reduced use of hospital services was a less certain, longer-term outcome. We therefore propose modifying it as fig. 2 shows.

Besides making a preliminary empirical evaluation, this study also contributes to knowledge by suggesting, in light of the above evidence, potential revisions to the DLC logic model to improve the intended outcomes in these and other settings. The revised logic model (figure 2) may be of practical use in guiding further development and applications of the DLC model, thereby contributing to strengthening the quality of the dementia care environment and the contribution of training to doing so.

### Research implications

We found *prima facie* evidence for parts of the DLC logic model, which we therefore conclude shows sufficient promise to merit fuller evaluation. Our results also expose the challenging case-mix, low quality of care and organisational instability of some care homes in the locality studied. The DLC intervention was developed to be transferrable and sustainable across the large number of care homes served by the NHS Trust. To implement other recognised practice improvement approaches across so many homes would not at present be feasible. Data limitations and the ‘on-balance’ character of some results mean that the present study should be regarded as initial exploratory research, containing findings (e.g. concerning recruitment, resources, methodologies, acceptability, outcome measures, potential effect sizes and contextual appropriateness [21]) relevant to designing any future RCT or quasi-experimental evaluation and showing how the elements of such a research study could work together. The case-mix which we found suggests either that QUALID is the more relevant quality measure for this care group, or that a much larger study would be needed if DEMQOL were a required outcome measure. A larger study would also be required for obtaining more certain results about DLC impacts on ambulance call-outs, hospital admissions and (not covered here) prescribing rates for anti-psychotics. The drop-out rates reported above also have implications for estimating sample sizes in further research. To examine changes in work routines directly, rather than indirectly via their assumed effects on well-being, would require using the DCM’s subsidiary ‘Personal Detractions’ and ‘Positive Events’, or similar, coding frames. Lastly this study highlights the question of what specific organisational conditions help or hinder PDSA cycles and staff development activities in having an impact upon work routines in care homes, a barely researched topic. An expanded ethnographic research component that included, for example, direct observation of the DLC training and the dementia champions’ meetings—in addition to the methods used in this study—would help answer questions around training and how its effects are achieved within care homes.

The DLC intervention, developed to strengthen the quality of the residential dementia care environment, combined a number of proven approaches including dementia training incorporating didactic and interactive elements [23, 58], continuous improvement processes [14–16], support through a community of practice [14], and recognition, coaching and reward of dementia care leadership and organisational culture change [10, 11]. This evaluation which tested the underlying DLC assumptions (logic model) about the relationship between these intervention components and the intended outcomes offers evidence for areas where this dementia care practice innovation may be further refined.

## Abbreviations

ADQ: Approaches to Dementia Questionnaire
DAS: Dementia Attitudes Scale
DCM: Dementia Care Mapping
DEMQOL: Dementia Quality of Life (questionnaire)
DLC: Dementia Learning Community
GDS: Global Deterioration Scale
NVQ: National Vocational Qualification
PDSA: Plan-Do-Study-Act (cycle)
QCF: Qualification and Credit Framework (for training)
QUALID: Quality of Life in Late-Stage Dementia (scale)
SNCW: Satisfaction with Nursing Care and Work (assessment scale)
TEP: Treatment Escalation Plan
WIB: Well/Ill Being (score)
